# HCV Induces Oxidative and ER Stress, and Sensitizes Infected Cells to Apoptosis in *SCID/Alb-uPA* Mice

**DOI:** 10.1371/journal.ppat.1000291

**Published:** 2009-02-06

**Authors:** Michael A. Joyce, Kathie-Anne Walters, Sue-Ellen Lamb, Mathew M. Yeh, Lin-Fu Zhu, Norman Kneteman, Jason S. Doyle, Michael G. Katze, D. Lorne Tyrrell

**Affiliations:** 1 Department of Medical Microbiology and Immunology, University of Alberta, Edmonton, Alberta, Canada; 2 Department of Microbiology, School of Medicine, University of Washington, Seattle, Washington, United States of America; 3 Department of Pathology, School of Medicine, University of Washington, Seattle, Washington, United States of America; 4 Department of Surgery, University of Alberta, Edmonton, Alberta, Canada; 5 Department of Pathology, Vernon Jubilee Hospital, Vernon, British Columbia, Canada; University of Florida, United States of America

## Abstract

Hepatitis C virus (HCV) is a blood-borne pathogen and a major cause of liver disease worldwide. Gene expression profiling was used to characterize the transcriptional response to HCV H77c infection. Evidence is presented for activation of innate antiviral signaling pathways as well as induction of lipid metabolism genes, which may contribute to oxidative stress. We also found that infection of chimeric *SCID/Alb-uPA* mice by HCV led to signs of hepatocyte damage and apoptosis, which in patients plays a role in activation of stellate cells, recruitment of macrophages, and the subsequent development of fibrosis. Infection of chimeric mice with HCV H77c also led an inflammatory response characterized by infiltration of monocytes and macrophages. There was increased apoptosis in HCV-infected human hepatocytes in H77c-infected mice but not in mice inoculated with a replication incompetent H77c mutant. Moreover, TUNEL reactivity was restricted to HCV-infected hepatocytes, but an increase in FAS expression was not. To gain insight into the factors contributing specific apoptosis of HCV infected cells, immunohistological and confocal microscopy using antibodies for key apoptotic mediators was done. We found that the ER chaperone BiP/GRP78 was increased in HCV-infected cells as was activated BAX, but the activator of ER stress–mediated apoptosis CHOP was not. We found that overall levels of NF-κB and BCL-xL were increased by infection; however, within an infected liver, comparison of infected cells to uninfected cells indicated both NF-κB and BCL-xL were decreased in HCV-infected cells. We conclude that HCV contributes to hepatocyte damage and apoptosis by inducing stress and pro-apoptotic BAX while preventing the induction of anti-apoptotic NF-κB and BCL-xL, thus sensitizing hepatocytes to apoptosis.

## Author Summary

Hepatitis C virus is a common cause of liver disease worldwide. The details of how HCV causes liver disease are not well understood. It has been thought that HCV infection does not kill liver cells directly, but indirectly by stimulating the immune system to kill HCV-infected liver cells. In this study we have used a mouse model that supports HCV infection and replication. These mice do not have an adaptive immune system. Despite the lack of an adaptive immune system, we have shown that HCV infection still leads to the death of infected liver cells. This study provides new insight into how HCV damages the liver in chronic HCV carriers.

## Introduction

Hepatitis C virus (HCV) is a positive strand RNA virus that belongs to the family *Flaviviradae*. HCV is a blood borne pathogen which is a major cause of liver disease worldwide, with an estimated 200 million people infected. It is estimated that 30% of chronically infected patients eventually develop progressive liver disease including cirrhosis and end stage liver disease [1]. HCV is now the leading indication for liver transplantation in North America [2]. Due the absence of a proofreading activity in the viral RNA polymerase, HCV has a high mutation rate that contributes to the genetic heterogeneity of the virus. Six different genotypes, and at least 52 subtypes have been described [3].

Chronic infection by HCV results in a highly variable disease course, and despite advances in the molecular virology of HCV, the factors involved in hepatocyte injury and the progression of liver disease remain unclear. The complexity of the host response has been examined by transcriptional profiling of liver biopsy samples from both chimpanzees and humans. These studies show that HCV induces genes involved in the interferon response, lipid metabolism, oxidative stress, and chemokines, as well as markers of inflammation [4],[5]. Such studies are hampered by the requirement that infected and uninfected hepatocytes come from different patients and also by the presence of an adaptive immune response in the patients. Gene expression profiles of human hepatocytes from *SCID/Alb-uPA* chimeric mice infected with HCV patient serum, control these variables [6]. Until recently it has been thought that HCV is a non-cytopathic virus and that hepatocyte damage in chronic HCV infection is due to HCV-specific adaptive immune responses [7],[8]. However, in *SCID* mice lacking an adaptive immune system, we observed induction of apoptosis in HCV infected mouse livers, similar to that seen in liver biopsies from HCV infected patients [6].

During HCV infection, hepatocyte apoptosis could be induced by immune attack on infected cells or directly by viral infection. It has been shown that hepatocyte damage can lead to apoptosis, which plays a role in the recruitment and activation of stellate cells and macrophages and the subsequent development of fibrosis [9],[10]. HCV infected patients have higher levels of immune related death ligands; TRAIL, TNF-α, FAS, and FASL are all elevated in HCV infected patients [11]–[13]. Increased expression of stimulators of apoptosis in HCV infected patients is tempered by hepatocyte insensitivity to death ligand mediated apoptosis. In hepatocytes death receptor mediated caspase-8 activation is weak, and thus they are inherently resistant to TNF-α and TRAIL killing [14],[15]. Hepatocytes are likely type II cells and can be sensitized to death ligand mediated apoptosis by caspase-8 induction with IFN-α (interferon) or toxins [16]–[18]. In addition, hepatocytes can be sensitized to both TRAIL and TNF-α induced apoptosis by inhibition of NF-κB activity [19]–[21]. Conversely, induction of NF-κB has been shown to inhibit TRAIL, TNF-α, and FAS mediated killing [22]–[24].

The induction of apoptosis directly by HCV remains controversial. Several HCV proteins have been proposed to have both pro- and anti-apoptotic effects [25]–[28]. It has been shown that expression of either the HCV genome or individual HCV structural proteins induces endoplasmic reticulum (ER) stress [27],[29] and the unfolded protein response (UPR), which can lead to apoptosis. However, HCV proteins have also been shown to modulate the UPR [30],[31]. It has also been proposed that HCV infection induces oxidative stress, which can enhance apoptosis [6],[32]. Expression of HCV core induces oxidative stress and expression of antioxidant genes [33],[34]. In addition, HCV patients have more DNA lesions produced by oxidative damage [35]. Oxidative stress leads indirectly from DNA damage to p53 induction, which can lead to activation of BAX and apoptosis [36],[37]. However, there are also reports of inhibition of p53 induced apoptosis by NS5A [38]–[41].

In this study, we used the mouse model for HCV infection in which severe combined immunodeficiency disorder (*SCID*) mice transgenic for an array of urokinase plasminogen activator (*uPA*) under control of the albumin (*Alb*) promoter are transplanted with human hepatocytes and then infected with HCV [6], [42]–[44]. We have previously compared HCV induced gene expression in chimeric mice infected with genotype 1a patient serum to uninfected controls containing human hepatocytes from the same donor [6]. There was evidence of activation of innate antiviral signaling pathways, induction of lipid metabolism genes, as well as signs of hepatocyte damage and an inflammatory response. To further reduce variation from HCV quasispecies present in the mice, in this study we have infected mice with the infectious clone HCV H77c [45],[46]. We confirmed the results of the previous study that used infectious patient serum, and to determine the cause of hepatocyte damage, we examined the expression HCV antigens and key proteins involved in the stress response and apoptosis using immunohistochemical and fluorescent confocal microscopy. We found that HCV infection correlated with increased levels of the ER chaperone GPR78/BiP, a key regulator of the unfolded protein response. In addition, levels of pro-apoptotic BAX were increased, while anti-apoptotic NF-κB and BCL-xL were decreased in HCV infected cells. Taken together these results indicate that ER stress induced by HCV combined with lower NF-κB and BCL-xL levels sensitizes hepatocytes to apoptosis.

## Results

**Figure 1:**
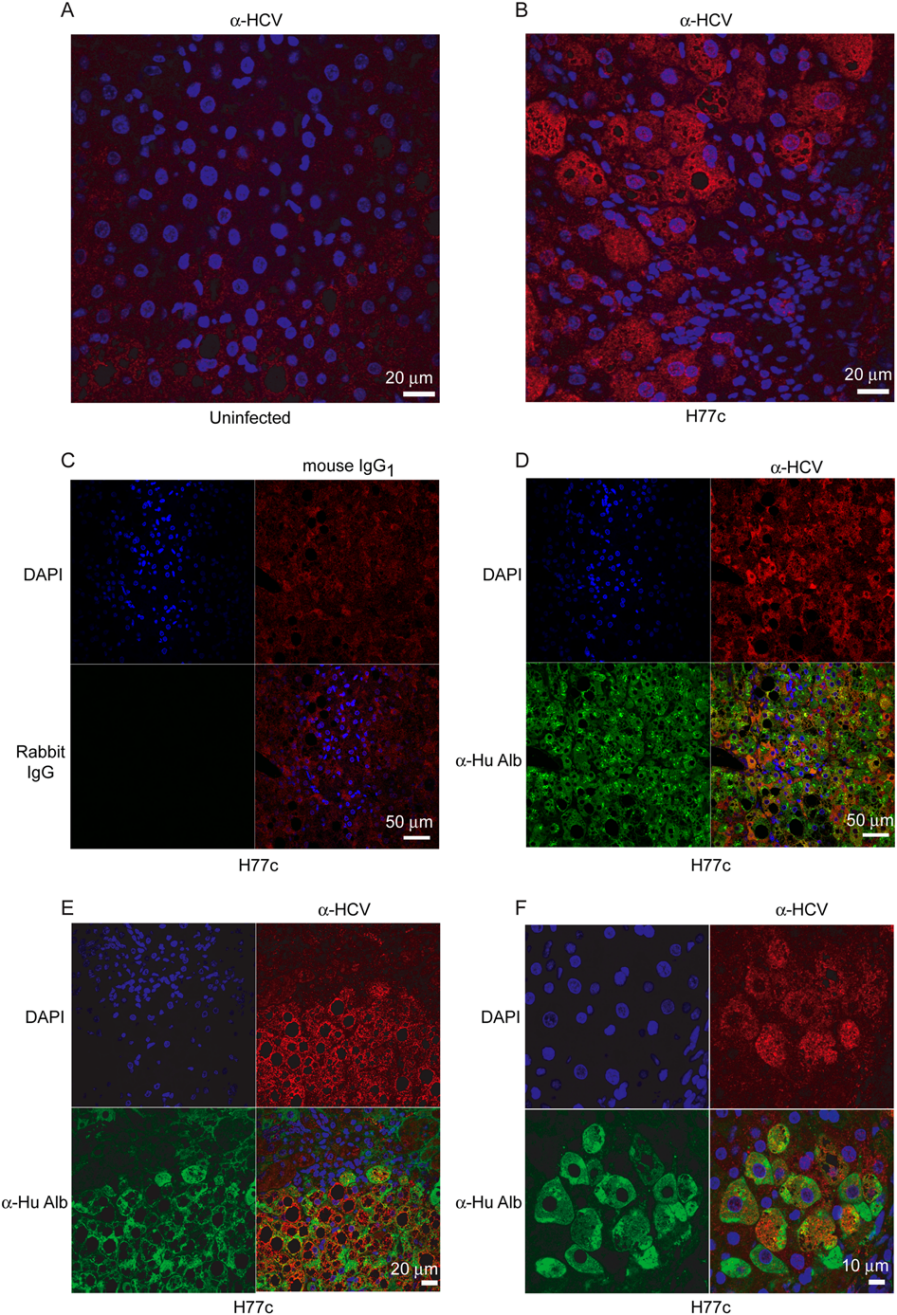
Confocal microscopy of uninfected and HCV H77c infected mouse livers stained with anti-HCV and anti-human albumin. Confocal microscopy was performed on either uninfected (A) or H77c infected (B–F) liver sections. Sections were either singly stained (A, B) using mouse monoclonal antibodies against HCV or, to differentiate between the human and mouse hepatocytes, rabbit anti human albumin antibodies in addition (D–F). Isotype controls (C) were also used to stain a serial section to panel D (See also figure S1 A, B). The nuclei were stained with DAPI (blue), and mouse antibodies were visualized using secondary goat anti-mouse poly-HRP antibodies followed by tyramide-TMR (red). The rabbit antibodies were visualized using secondary goat anti-rabbit-alexa 488 (green) antibodies. Junctions between human and mouse cells is shown in two magnifications in panels E and F.

**Figure 2:**
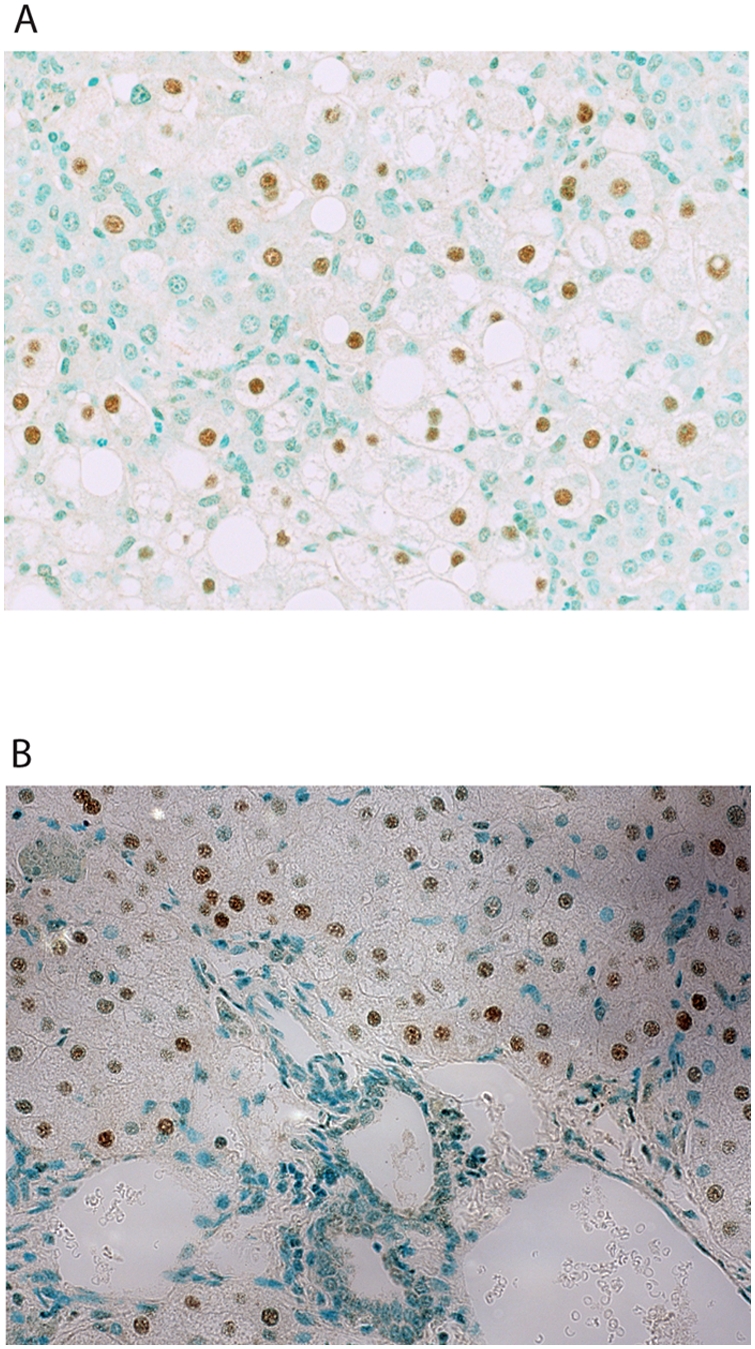
In situ hybridization with human specific *Alu* DNA oligonucleotides. In situ hybridization using human *Alu* DNA probes, with the nuclei were counter stained using methyl green, showing a lobular region (A), and portal triad (B). Magnification ×400.

Previous studies indicated HCV infection in chimeric mouse livers was restricted to the human hepatocytes [44]. We confirmed this by performing immunofluorescent confocal microscopy on uninfected and HCV H77c infected mouse livers with antibodies specific for the HCV NS3 protease and human albumin (Figure 1 and S1A–B). Only liver sections infected with HCV H77c stained with HCV specific antibodies, and this was restricted to hepatocytes that also were also stained with antibodies specific for human albumin. Since liver consists of a mix a hepatocytes and adventitial cells, and albumin only stains hepatocytes, there was the possibility that some human cells other than hepatocytes also colonized the mouse liver. Therefore we wished to examine whether any of the adventitial cells were also human. We performed *in situ* hybridization using probes specific for human *Alu* repeats on chimeric mouse livers (Figure 2). Only the hepatocytes were stained, indicating all of the adventitial cells in chimeric mouse livers were of mouse origin. This, and the elimination of a small percentage of mouse sequences that cross-hybridized to the human arrays, ensured that the transcriptional profiling reflects only the processes occurring in human hepatocytes.

**Figure 3:**
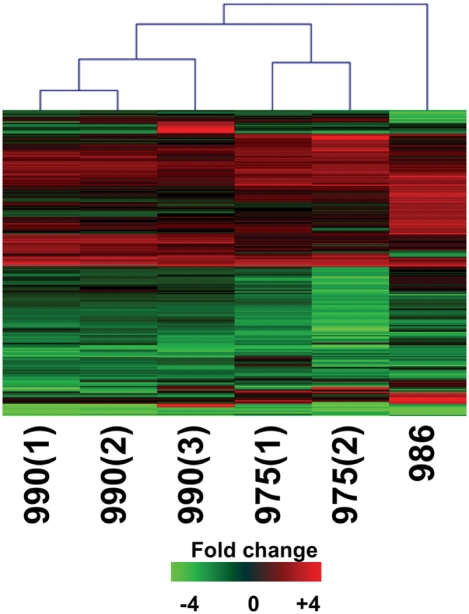
Global gene expression profiles of HCV (H77c 1a)-infected mice. Two-dimensional hierarchical clustering was performed using Resolver System software with an agglomerative algorithm, complete link heuristic criteria, and Euclidian correlation metric. Each column represents gene expression data from an individual experiment (either individual HCV-infected mouse or individual liver sample) and the cluster represents genes that showed a >2-fold change (P value<0.05) in at least 1 experiment. Genes shown in red were up-regulated; genes shown in green down-regulated and genes in black indicate no change in expression in HCV-infected tissue relative to a pool (3 individual animals) of donor-matched uninfected tissue. Animal 990 was infected with H77c (+) serum. Animal 975 received 100 µg of H77c RNA by intrahepatic injection while animal 986 received 100 µg of H77c RNA containing the GDD-AAA mutation in the NS5B coding region.

Transcriptional profiling was performed on mRNA samples isolated from three HCV-infected animals and from uninfected controls. All animals contained hepatocytes from a single donor. The serum HCV titers, liver viral loads, and the length of time infected are given in Table 1. The experiments included three liver samples from an animal infected with H77c (+) serum (990), two samples from an animal inoculated intrahepatically with H77c RNA (975), and a single experiment with liver tissue from an animal inoculated intrahepatically with H77c RNA containing a mutation in the active site of the NS5B polymerase (986). Four samples from three separate animals were pooled to serve as the uninfected control. Because each pair of mice contained hepatocytes from the same donor, changes in gene expression should mainly be induced by the HCV infection and be independent of host variation. Consistent with previous studies, the effect on host gene expression by HCV infection was not extreme, 766 genes showed a 2-fold or higher change in expression (P value≤0.05) in at least one experiment (Figure 3). The grouping of experiments by the clustering algorithm suggested that the effect on host gene expression was very similar among individual pieces of liver from the same animal. Importantly, the global gene expression profiles in the animals infected with H77c (+) serum (990) and H77c RNA (975) were also very similar. This suggests that the source of HCV inoculum does not significantly impact the host transcriptional response to infection. Interestingly, the animal inoculated with H77c RNA encoding an inactive NS5B polymerase also showed a similar host response. While it was expected that the mouse inoculated with the replication defective HCV RNA might show activation of dsRNA and RIG-I signaling pathways similar to replicating virus [47],[48], we expected substantial differences in the overall host response 47 days after RNA administration. This mouse showed no detectable HCV RNA in the serum at the time of sacrifice and no HCV RNA was detected in the sample used for microarray analysis.

**Table 1:** Serum and Liver Viral Titers.

Mouse	Serum titer^a^ (copies/ml)	Average Liver load (copies/µg Human RNA)	Length of Infection
**Naïve:**			
** 005**	undetectable	undetectable	Age match 30 days
** 963**	undetectable	undetectable	Age match 30 days
** 992**	undetectable	undetectable	Age match 47 days
** 996**	undetectable	undetectable	Age match 47 days
**986-AAA**	undetectable	undetectable	47 days
**638-1a Patient serum**	4.7×10^5^	1.6×10^5^	25 days
**975-RNA**	1.3×10^6^	5.3×10^4^	47 days
**985-RNA** b	1.9×10^2^	1.8×10^2^	47 days
**990-H77c serum**	9.5×10^6^	3.8×10^5^	47 days

a The detection limit for this assay is 255 copies/ml.

b Although the serum titer was below the limit of detection at the time of death, animal 985 was infected because at days 21 and 33 post infection the titers were 1×10^3^ and 7.7×10^3^, respectively.

**Figure 4:**
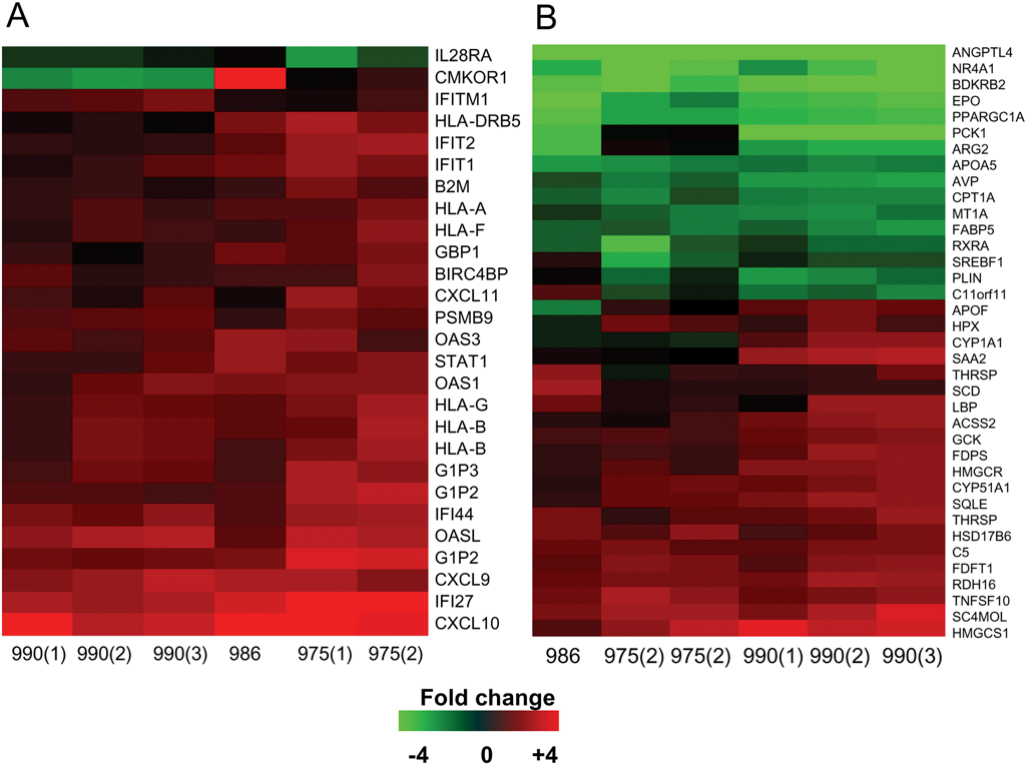
Expression of IFN-inducible and lipid metabolism genes in human liver tissue from HCV-infected mice. The gene sets were IFN-inducible (A), or involved in lipid metabolism (B). Two-dimensional hierarchical clustering was performed using Resolver System software with an agglomerative algorithm, complete link heuristic criteria, and Euclidean correlation metric. Each column represents gene expression data from an individual experiment (either individual HCV-infected mouse or individual liver sample). Genes were selected as at least 2-fold regulated (P value<0.05) in at least 1 experiment. Genes shown in red are up-regulated and genes shown in green are down-regulated in HCV-infected tissue relative to donor-matched uninfected tissue, while black indicates no change in gene expression. Animal 990 was infected with H77c (+) serum. Animal 975 H77c RNA by intrahepatic injection while animal 986 received H77c RNA containing the GDD-AAA mutation in the NS5B coding region.

Infection with HCV H77c activated innate antiviral signaling pathways, as indicated by the induction of interferon-stimulated genes (ISGs) (Figure 4A). In general, the induction was similar among all three infected animals. However, there does seem to be a slightly higher induction of ISGs in the animal (975) inoculated intrahepatically with wild-type H77c RNA relative to the animal (990) inoculated with H77c (+) serum, which is likely due to the high level of naked RNA injected directly into the liver of this animal (100 µg, 2×10^13^ copies). This increased response relative the animal inoculated with serum (990) was not observed in the animal (986) injected with H77c RNA containing inactive NS5B, indicating that part of the increased response in animal 975 might be due to replication of the inoculated HCV RNA. In our previous study of mice infected with HCV-positive patient sera the magnitude of induction of ISGs varies among mice containing hepatocytes from different donors. Comparison of the gene expression data from HCV H77c-infected mice with that from the initial study indicate the induction of ISGs in the H77c-infected mice is relatively weak. Consistent with what was observed in animals with a weak IFN response in the initial study, regulation of numerous genes associated with lipid metabolism were observed in the HCV H77c-infected mice (Figure 4B). These included genes involved in cholesterol and fatty acid biosynthesis, β-oxidation and peroxisome proliferation. There did not appear to be any significant differences due to inoculum source. Interestingly, the animal that received replication incompetent H77c RNA also showed some regulation of these genes, although at a lower magnitude. The fact that this mouse shows any changes at all, in the absence of viral replication, may be because injection of viral RNA from a positive-sense RNA virus likely results in the synthesis of viral proteins.

**Figure 5:**
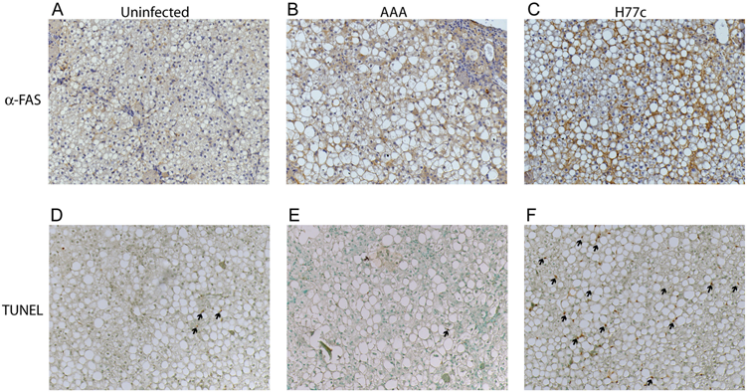
Immunohistochemical analysis of FAS expression and TUNEL reactivity. Liver sections from PBS injected (A and D), replication deficient RNA injected (B and E) and HCV H77c infected (C and F) donor matched chimeric mice were stained using rabbit anti-FAS (A–C), developed using the Vecastain ABC kit and counterstained using haematoxylin. Isotype controls were negative and are shown in Figure S1C–D. TUNEL (D–F) was performed using the Apoptag Plus Peroxidase In Situ Apoptosis Detection kit and the nuclei were counterstained with methyl green. Magnification ×100. Arrows indicate TUNEL reactive nuclei. In panel F, arrows indicate only some of the TUNEL reactive nuclei.

Consistent with the up regulation of genes involved in oxidative stress seen in this and previous expression array studies, histological analysis revealed signs of hepatocyte damage in the human hepatocytes of HCV infected chimeric mice (Table 2). Steatosis was apparent in the majority of the human hepatocytes regardless of infection. However, there were significant differences in the histology between the animals inoculated with HCV RNA and naive animals. Increased hepatocyte ballooning and lobular inflammation were associated with HCV-infection. Staining of sections with the antibody F4-80 (anti CD68) revealed that the lobular inflammation was due to infiltration of monocytes and macrophages (not shown). A particularly intriguing observation was the presence of apoptotic hepatocytes in HCV-infected animals originally detected as caspase-3 activation and quantitation of apoptotic bodies [6]. We also observed apoptosis in H77c-infected mice by TUNNEL assay (Figure 5D–F). Apoptosis was absent in the animal inoculated with the replication defective H77c-AAA mutant. This suggests that apoptosis observed in HCV-infected mice is dependent upon active HCV replication. The expression of genes associated with cell death was analyzed to gain further insight into possible mechanisms of apoptosis. While there was regulation of cell-death related genes in HCV H77c infected animals, the number of genes affected is small (data not shown). This is perhaps not surprising given the low percentage of hepatocytes that are actually undergoing apoptosis. Quantitation of the TUNEL data in Table 2 (average 723 cells/field) revealed on average 5% of cells undergoing apoptosis in infected mice.

**Table 2:** Immunohistological Evaluation of Liver Sections.

Mouse	Fat^a^	Balloon^b^	Grade^c^	Stage^c^	FAS^d^	LAB^e^	Caspase^d^	Tunel^f^
**Naive:**								
** 005**	0	0	0(P0 L0)	0	2	0.2	0	3±2
** 963**	2	1	0(P0 L0)	0	1	0	0	8±3
** 992**	3	0	2(P0 L2)	0	3	0	2 m	8±3
** 996**	2	0	2(P0 L2)	0	2	0	2 m	11±2
**986-AAA**	2	2	3(P0 L3)	0	3	0	0	10±4
**638-1a Patient serum**	0	3	2(P0 L2)	0	4	0.4	2 m	18±2
**985-RNA**	3	3	3(P0 L3)	0	3	1.6	3 m, h	50±14
**975-RNA**	2	3	3(P0 L3)	0	4	4	3 m, h	26±13
**990-H77c serum**	3	2	2(P0 L2)	0	4	0.4	2 m	50±13

a The degree of fatty change was scored as 0 (<5%), 1 (6–33%), 2 (34–66%) 3 (>66%).

b Hepatocyte ballooning was scored on a scale of 0–4 where 0 is none and 4 is many.

c Grade and Stage were scored using the Batts and Ludwig criteria.

d FAS and caspase staining was scored semi-quantitatively where 0 = none, 1 = focal weak, 2 = diffuse weak; 3 = diffuse weak and focal strong; 4 = diffuse strong. For caspase staining, m is macrophages and h is hepatocytes.

e The lobular apoptotic body count is an average of 5 fields counted at magnification ×100.

f The number of Tunel positive nuclei is an average of 15 fields at magnification ×200.

**Figure 6:**
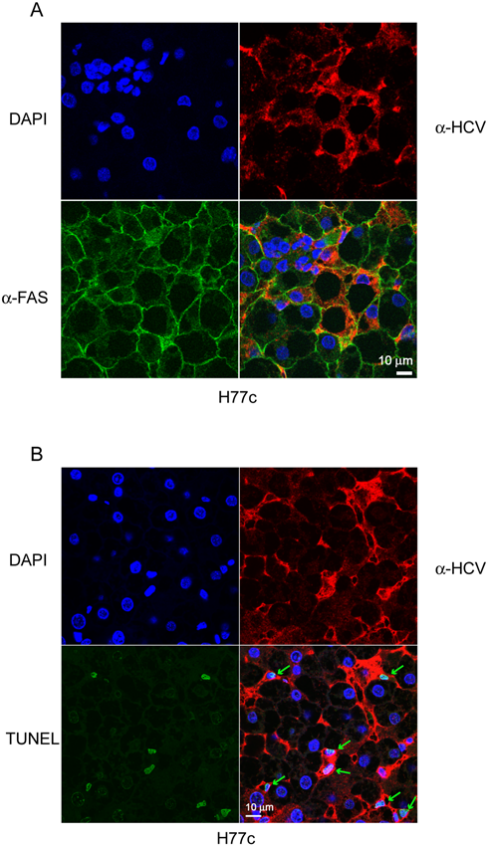
Confocal microscopic analysis of HCV antigen expression and either FAS expression or TUNEL reactivity. Staining for FAS (A) or TUNEL reactivity (B) was performed as described in Materials and Methods, using primary mouse anti-HCV and either rabbit anti-human FAS (A), or for TUNEL, FITC labeled dUTP and terminal deoxynucleotidyl transferase (B). The secondary antibodies were goat anti mouse poly-HRP, and for FAS staining, goat anti rabbit Alexa-488 (green). HRP was developed using the TSA plus fluorescence system with tyramide-TMR (red). Nuclei were stained using DAPI (blue). Arrows indicate TUNEL reactive nuclei.

To further investigate the mechanism of increased apoptosis associated with HCV infection, we examined FAS expression on liver sections from infected and uninfected mice and compared this to liver sections subjected to TUNEL analysis. Similar to what has been seen in mice infected with patient serum and in patient biopsies [6], there was increased FAS staining in infected compared to donor matched uninfected mice (Figure 5A–C). As can be seen in Figure 5 and at higher magnification in Figure S2, there was strong FAS reactivity in a majority of the human cells in infected mice, however TUNEL positive nuclei were seen only in a small proportion of human cells (Figure 5D–F). Interestingly, although staining was not as intense, there was an increase in FAS staining in the mouse inoculated with the H77c-AAA mutant, without a correlative increase in the TUNEL positive nuclei (Table 2). This suggests that increased FAS expression is not the only factor required for induction of apoptosis and TUNEL reactivity. We next investigated the correlation between HCV infection, and either FAS expression, or TUNEL reactivity. When we stained liver sections with FAS- and HCV-specific antibodies (Figure 6A), we found that expression of FAS does not depend on the presence of HCV in the cells, but is a host reaction to infection of neighbouring cells. However, we cannot rule out the possibility that only a portion of cells in the liver express enough HCV antigen to be detected using this antibody. When we subjected liver sections to a fluorescent TUNEL assay and then stained them with HCV specific antibodies, we found that all of the TUNEL positive cells in areas populated by human hepatocytes also stained with HCV specific antibodies (Figure 6B). The exception was that some murine Kupffer cells which also contained multiple TUNEL positive nuclei. Thus, HCV replication seems to be required for hepatocyte apoptosis. This is unlike FAS expression, which could be induced by HCV replication in neighbouring cells.

To further investigate the relationship between HCV infected cells and apoptosis we performed immunohistochemistry and fluorescent confocal microscopy using antibodies for key proteins involved in apoptosis. It has been proposed that HCV induces oxidative stress [6],[32]. Oxidative stress can lead to the induction of p53 and BAX, both of which can translocate to the mitochondria and induce apoptosis [36],[37]. We performed immunohistological staining for p53 on infected and uninfected liver sections and found no evidence for p53 induction in either the cytoplasm or nucleus or its translocation to the mitochondria except in one infected animal where very few cells appeared to have up-regulated p53 (data not shown).

**Figure 7:**
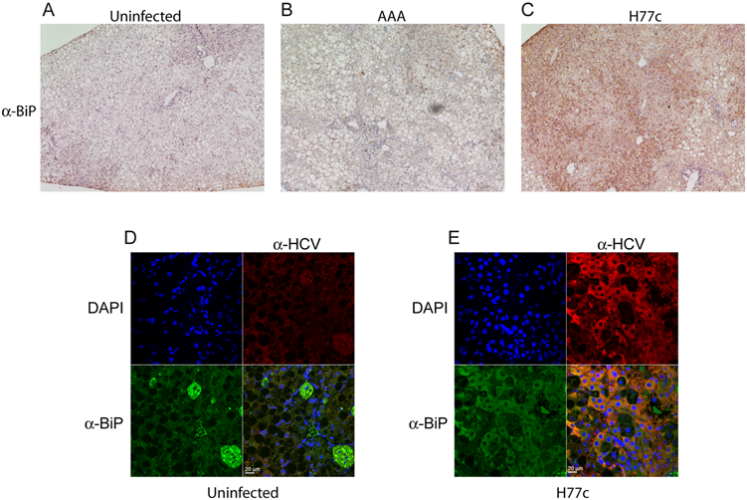
Immunohistochemical and confocal microscopic analysis of BiP/Grp78 expression. BiP expression was examined by either immunohistochemistry (A–C) or confocal microscopy (D–E). Liver sections from PBS injected (A and D), replication deficient RNA injected (B) and HCV H77c infected (C and E) donor matched chimeric mice were stained using goat anti-BiP/Grp78 alone (A–C) and developed using the Vecastain ABC kit. Magnification ×100. Isotype controls are shown in Figure S1 E–F and are negative. For confocal microscopy (D and E), primary antibodies were goat anti-BiP/Grp78 with mouse anti-HCV, and the secondary antibodies were donkey anti-goat alexa 488 (green), and donkey anti-mouse biotin followed by avidin-HRP (Vector laboratories). The peroxidase was developed as before using tyramide-TMR (red). Nuclei were stained using DAPI (blue).

**Figure 8:**
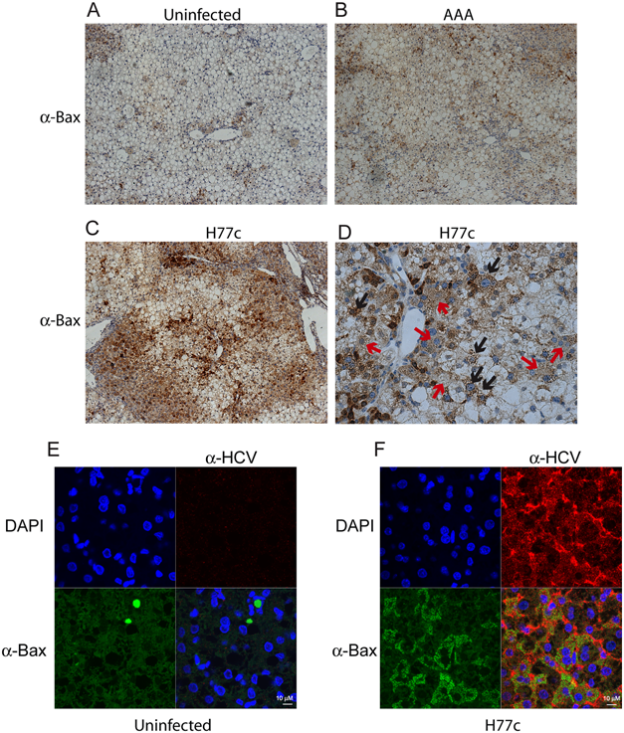
Immunohistochemical and confocal microscopic analysis of BAX expression. BAX expression was examined by either immunohistochemistry (A–D) or confocal microscopy (E–F). Liver sections from PBS injected (A and E), replication deficient RNA injected (B) and HCV H77c infected (C, D and F) donor matched chimeric mice were stained using rabbit anti-BAX alone (A–D) and developed using the Vecastain ABC kit. Magnification ×100 (A–C). A magnification ×400 view is shown in D with red arrows indicating cells diffuse BAX, and black arrows indicating cells with punctate BAX. Isotype controls are shown in Figure S3 and are negative. For confocal microscopy, primary antibodies were rabbit anti-BAX with mouse anti-HCV, and the secondary antibodies were goat anti-rabbit alexa 488 (green), with goat anti-mouse poly-HRP, developed using tyramide-TMR (red). Nuclei were stained using DAPI (blue). Comparison of a field from an area of liver containing only mouse cells with an HCV infected human area is shown in Figure S3.

Expression of HCV structural proteins has been shown to induce ER stress [27],[49], which can induce the oligomerization and translocation of BAX/BAK to the mitochondria. To examine the role of ER stress in HCV induced apoptosis, we compared the expression of the ER chaperone GRP78 (BiP) in uninfected and H77c infected mice by immunohistochemistry (Table 3 and Figure 7A–C) and by fluorescent confocal microscopy (Figure 7D, E). We found higher levels of BiP in infected mice, and that BiP expression correlated with HCV infection. Additionally, BiP and HCV seemed to co-localize, consistent with replication of HCV on the ER. The ER chaperone BiP is a key sensor in the unfolded protein response (UPR); it maintains ER membrane signal proteins in inactive states. It has been shown that BAX and BAK interact directly with one of these membrane signal proteins, IRE1, and are essential for IRE1 activation [50]. Extensive or prolonged ER stress initiates apoptosis through activation of BAX/BAK. We therefore examined the expression of BAX in liver sections (Table 3 and Figure 8) and found BAX was also overexpressed in HCV infected livers. BAX is normally diffusely expressed throughout the cell, however when activated it translocates to the mitochondria and appears as a granular staining pattern. We found that both patterns of staining were elevated in HCV infected livers (Figure 8A–D). Figure 8D shows both the intense granular staining pattern as indicated by the black arrows and the less intense cytoplasmic staining indicated by the red arrows. The large granular staining pattern correlated with HCV infection (Figure 8F). It is worth noting that not all cells that stained positive for HCV also showed activated BAX. The number of cells staining for activated BAX approximately correlated with the number of TUNEL positive nuclei. The elevation of both BiP and BAX in the absence of increased levels of p53 suggests that ER rather than oxidative stress leads to BAX activation, however additional mechanisms of BAX activation by oxidative stress cannot be eliminated.

**Table 3:** Immunohistochemical Evaluation of Liver Sections Stained with Bip/GRP78 or Bax.

Mouse	BiP/GRP78	BAX	Punctate BAX^b^
	Cell number^a^ (Intensity)	Cell number^a^ (Intensity)	Cell number
**Naive:**			
** 005**	1 (1)	1 (1)	1
** 963**	2 (2)	1 (1)	1
** 992**	1 (1)	2 (1)	2
** 996**	1 (3-infiltrate)	0 (0)	0
**638-1a Patient serum**	2 (2)	2 (2)	2
**986-AAA**	1 (3-infiltrate)	2 (1)	0
**985-RNA**	3 (1)	3 (3)	3
**975-RNA**	2 (2)	2 (2)	3
**990-Serum**	3 (2)	2 (2)	2

a Staining was scored semi-quantitatively where 0 is no staining 1 (1–15%), 2 (16–30%), and 3 (31–50%), and the intensity of staining was scored on a scale of 0–3, where 0 is no staining and 3 is intense staining.

b Activated BAX has a distinct punctate staining which is easily distinguished form inactive BAX, 0 is no staining, 1 (1–5%), 2 (5–10%), and 3 (10–15%). Activated Bax has a uniform intense staining and so no intensity score was given.

**Figure 9:**
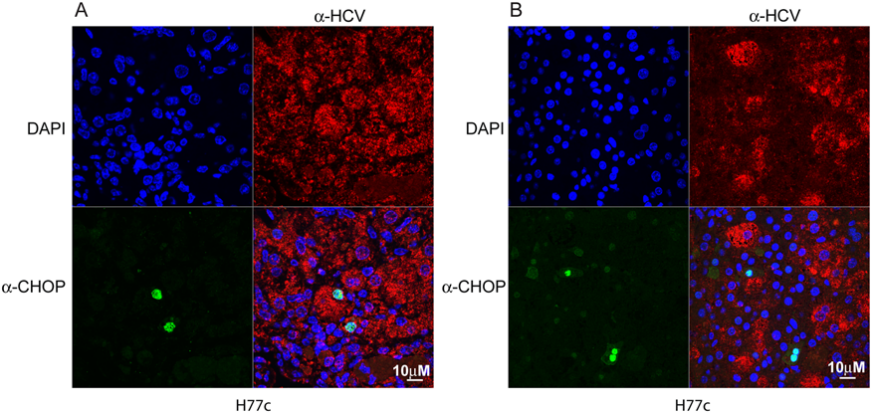
Confocal microscopy of HCV and CHOP/GADD153 in predominately infected or uninfected areas of HCV infected mice. Panel (A) shows an area from an HCV infected liver that contains mostly infected cells. Panel (B) shows an area from an HCV infected liver that contains mostly uninfected cells. Liver sections from HCV H77c infected mice were stained using rabbit anti-CHOP and mouse anti-HCV antibodies, and the secondary antibodies were goat anti rabbit alexa 488 (green) and goat anti mouse poly-HRP, which was developed using tyramide-TMR (red). Nuclei were stained using DAPI (blue).

In cells under ER stress, BiP preferentially binds to malfolded proteins, releasing IRE1, PERK, and ATF6, activating downstream effectors which induce transcription of alarm and adaptation genes including BiP itself and GADD153 (CHOP) [51]. When the UPR is overwhelmed, apoptosis is induced by a number of molecules including CHOP, which translocates to the nucleus and blocks transcription of BCL-2, [52] an inhibitor of BAX/BAK. To examine whether the ER stress found in infected livers overwhelmed the unfolded protein response, indicated by translocation of CHOP to the nucleus, we performed immunofluorescent confocal microscopy with anti-CHOP and anti-HCV antibodies (Figure 9). Because we found very few nuclei that stained positively for CHOP and these did not correlate with the staining by HCV specific antibodies, in Figure 9 both panels are from infected mice; panel A shows a predominately infected area, and panel B shows a predominantly uninfected area with only a few infected cells. CHOP can be elevated in both infected and uninfected cells indicating that HCV infection does not overwhelm the UPR. This may explain why we do not see expression of ER stress genes in the microarray analysis.

**Figure 10:**
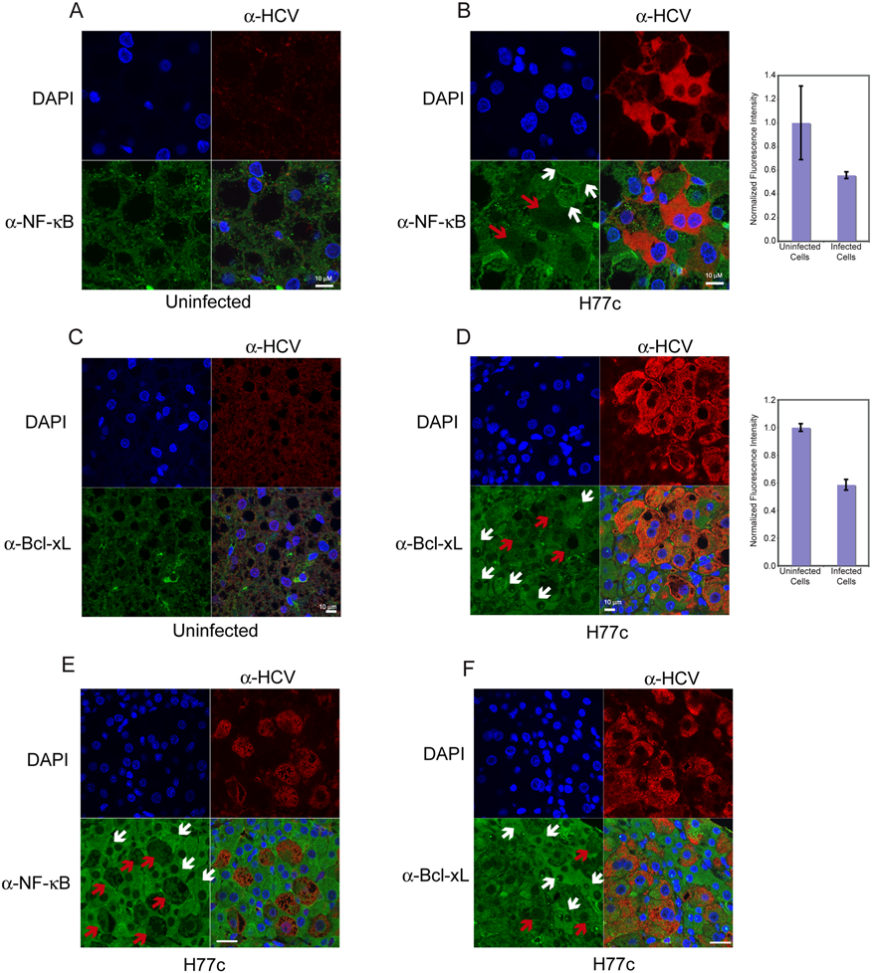
Confocal microscopy of HCV and either NF-κB p65 or BCL-xL. Liver sections from PBS injected (A and C) and HCV H77c infected (B and D) chimeric donor matched mice were stained using anti-NF-κB p65 (A and B) or BCL-xL (C and D) antibodies. Panels E and F are from non-donor matched infected mice stained with anti-NF-κB p65 (E) or BCL-xL (F) Primary antibodies were rabbit anti-NF-κB or rabbit anti BCL-xL with mouse anti-HCV, and the secondary antibodies were goat anti-rabbit alexa 488 (green), with goat anti-mouse poly-HRP developed using tyramide-TMR (red). Nuclei were stained using DAPI (blue). Red arrows indicate infected cells and white arrows indicate uninfected cells. The right hand panels are quantitation of p65 and BCL-xL levels in uninfected and HCV infected cells in HCV infected livers. For p65, 6 fields (61 infected and 59 uninfected cells) were quantified using Metamorph software. For BCL-xL, 4 fields (44 infected and 43 uninfected cells) were quantified. To compare cells from several fields, the average of the uninfected cells in a single field was arbitrarily set to 1 and the infected cells in that field were scaled appropriately. Isotype controls and comparison of a field from an area of liver containing only mouse cells with an HCV infected human area are shown in Figure S4.

In addition to pro-apoptotic proteins, we also examined key inhibitors of apoptosis. Both CHOP and NF-κB are both activated by ER stress [53],[54], but have opposing effects; CHOP is pro-apoptotic while NF-κB is anti-apoptotic. NF-κB is activated in response to a myriad of other stimuli, at least one of which is inhibited by HCV [55]. BCL-xL inhibits the apoptosis induced by BAX, and its transcription is activated by NF-κB [56]. Overall, when HCV infected livers were compared with uninfected livers, the levels of both NF-κB and BCL-xL appeared to be elevated in the infected liver, consistent with expression analysis, which indicated that NF-κB levels are elevated by HCV infection. However, when HCV infected cells are compared to uninfected cells within HCV infected livers, we found that total levels of NF-κB p65 expression was lower in HCV infected cells than in surrounding uninfected cells (Figure 10A–B and E and S4). Quantitation of total p65 fluorescence from uninfected and infected cells in 6 fields from infected livers revealed that p65 levels in infected cells were approximately half that in uninfected cells. The average fluorescence of uninfected cells in a field was arbitrarily set to 1. Consistent with reduced expression of NF-κB, the expression of BCL-xL was also lower in cells that stained with HCV specific antibodies (Figure 10C–D and F). Quantitation of total BCL-xL levels in infected livers also revealed that levels of BCL-xL in infected cells were approximately half of that in infected cells.

## Discussion

The course of HCV pathophysiology is extremely variable, as a result of complex interactions between viral variants and the host's innate and adaptive immune systems. We have used SCID/Alb-uPA mice that have chimeric human and mouse livers to examine the processes that occur in hepatocytes in response to HCV infection. Previous studies have shown that the transcriptional response HCV infection in mice is similar to that of humans and chimpanzees, with the exception of immune cell markers, which are absent in SCID mice. To further simplify the host response to infection, we infected mice with RNA from the HCV clone H77c [45],[46]. We found the same induction of interferon response genes and changes in expression of genes involved in lipid metabolism seen in earlier studies. This has been postulated to lead to the generation of oxidative stress [6],[57], and it has been shown that both oxidative stress and ER stress can lead to apoptosis [27],[29],[58],[59]. As well, apoptosis is one of the factors in the induction of fibrosis, which can culminate in cirrhosis [9],[10],[60],[61]. Interestingly, in the absence of an adaptive immune system, there was evidence for the induction of apoptosis in HCV infected mice. This was restricted to HCV infected cells despite increased FAS expression on both infected and uninfected human hepatocytes of infected animals. This generalized FAS expression may be a consequence of the interferon response occurring throughout the liver [6] since FAS/FASL are among those mediators of apoptosis that are also interferon response genes [62].

We therefore examined processes leading to apoptosis that we thought were likely to be affected by HCV in infected cells. Oxidative stress generated by HCV induced lipid metabolism, and ER stress generated by HCV replication and protein translation in and on the ER were both potential candidates. Hepatocytes are type II cells that contain low levels of caspase 8 and therefore require activation of the mitochondrial apoptosis amplification pathway to initiate apoptosis. This can be blocked by over expression of BCL-2 or BCL-xL. Mitochondria seem to be the site where the antiviral interferon response and apoptotic signals are integrated; recently it has been shown that the mitochondrial signaling molecule interferon promoter stimulating factor-1 (IPS-1) is cleaved during apoptosis and cleavage can be blocked by overexpression of BCL-xL [63]. In addition, both the response to oxidative stress and the response to ER stress converge at the mitochondrion; p53 activated by oxidative stress stimulates the oligomerization of BAX [36], and BiP responding to ER stress releases IRE-1 to which BAX is bound. BAX has been shown to be required for both IRE-1 activation and for apoptosis initiated by ER stress [50],[51]. Since oxidative stress can lead to p53 induction, Bax activation and apoptosis [36],[37], we examined p53 localization and levels and found that they were not affected by HCV infection. This may be due to NS5A mediated inhibition of the mitochondrial translocation, apoptosis inducing, and DNA binding activities of p53 [38]–[41]. ER stress can lead to induction of the UPR, and activation of BiP, CHOP, BAX and apoptosis. Consistent with the generation of ER stress by HCV we found that induction of the ER chaperone BiP and pro-apoptotic BAX correlated with HCV expression, but there was little translocation of CHOP/GADD153 to the nucleus, which indicated that the UPR was not overwhelmed.

**Figure 11:**
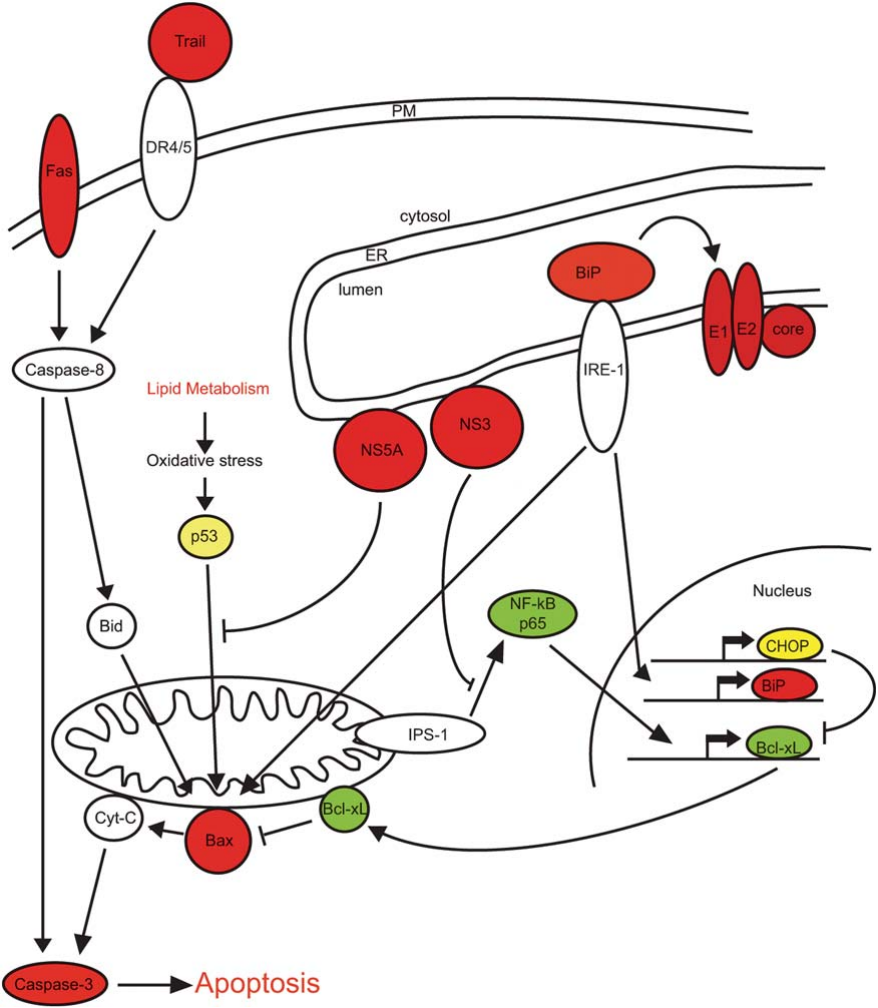
HCV sensitizes infected hepatocytes to apoptosis. The pathways that link oxidative and ER stress with apoptosis are shown, as is a potential interaction between the host cell response and apoptosis. In hepatocytes the levels of caspase 8 are low and induction of apoptosis requires the mitochondrial amplification loop, the convergence of the stress pathways at the mitochondria and low levels of NF-κB and BCL-xL sensitize hepatocytes to apoptosis. Proteins that we have shown are elevated in HCV infected cells are shown in red, those that do not change are shown in yellow and those that are decreased are shown in green.

In hepatocytes, it appears that NF-κB is one of the key determinants of whether apoptosis is induced in response to death ligands [19]–[24],[64]. In evading of the interferon response, HCV inhibits the activation of NF-κB; inhibition of the TLR-3 and RIG I pathways by cleavage of TRIF and IPS-1/MAVS/VISA/Cardif by the HCV NS3/4A protease, inhibits NF-κB and IRF-3 phosphorylation preventing nuclear translocation in response to RIG-1 activation by viral RNA [65]–[68]. In addition, there are a number of other reports that HCV modulates NF-κB activity [69]–[72]. Consistent with the reports of inhibition of NF-κB we found that total levels of NF-κB p65 were lower in HCV infected cells. Furthermore consistent with the transcriptional regulation of BCL-xL by NF-κB, we found that total levels of BCL-xL were lower in HCV infected cells. In conclusion, we propose a model (Figure 11) where HCV induces both ER stress and oxidative stress in infected cells, and activates pro-apoptotic Bax while it prevents induction of anti-apoptotic BCL-xL thus sensitizing HCV infected cells to apoptosis which may be mediated by death receptors and ligands, for example FAS and TRAIL (TNFSF10-Figure 4B). A combination of induction of pro-inflammatory chemokines (Figure 4A) and cross talk between human and mouse chemokines and their receptors may lead to a situation similar to that in patients; inflammation, which in turn stimulates release of pro-inflammatory cytokines and effector molecules such as TNF-α and FasL (which in these mice may be released by macrophages and NK cells), creating the circle of hepatocyte damage and repair that is a hallmark of HCV infection.

## Materials and Methods

### Transplantation and infection of chimeric SCID/Alb-uPA mice

All mice were housed VAF and treated according to Canadian Council on Animal Care guidelines. Experimental approval came from the University of Alberta Animal Welfare Committee, and human hepatocytes were obtained following informed consent of all donors with ethics approval from the University of Alberta Faculty of Medicine Research Ethics Board. Animals were transplanted with freshly isolated human hepatocytes [42],[44],[73]. Eight weeks after transplantation mice with human α-1antitrypsin (hAAT) levels [42] greater than 100 µg/mL were injected intrahepatically (*ih*), with 50 µg of *in vitro* transcribed H77c RNA [45] into each of 2 red liver nodules (presumed to be human hepatocytes). As a negative control, mice were injected *ih* with non-replicative H77c RNA in which NS5B polymerase active site residues GDD (amino acids 2736–2738) have been changed to AAA (H77c-AAA). Passage of H77c virus was done by *ih* inoculation of naive mice with 50 µL of serum obtained from mice infected by H77c RNA. One mouse was infected by *ih* inoculation with patient serum for histochemical comparison. Serum samples were taken at various time points after inoculation and HCV RNA was quantified. Animals were infected for 25 or 47 days and dissection of mouse livers, isolation of RNA, genomic DNA, and ratio of human to mouse cells in each sample was performed as previously described [43]. The serum HCV titers, liver viral loads and the length of time infected are given in Table 1. The plasmid for *in vitro* transcription was pCV H77c and was a gift from Dr. Jens Bukh.

### Microarray expression format and data analysis

The purity of human hepatocytes was greater than 70% in all samples used for microarrays. Microarray format, protocols for probe labeling, and array hybridization are described at http://expression.microslu.washington.edu. Briefly, a single experiment comparing two mRNA samples was done with four replicate Human 1A (V2) 22K oligonucleotide expression arrays (Agilent Technologies) using the dye label reverse technique. This allows for the calculation of mean ratios between expression levels of each gene in the analyzed sample pair, standard deviation and P values for each experiment. Spot quantitation, normalization and application of a platform-specific error model was performed using Agilent's Feature Extractor software and all data was then entered into a custom-designed database, Expression Array Manager, and then uploaded into Rosetta Resolver System 4.0.1.0.10 (Rosetta Biosoftware, Kirkland, WA) and Spotfire Decision Suite 7.1.1 (Spotfire, Somerville, MA). Data normalization and the Resolver Error Model are described on the website http://expression.microslu.washington.edu. This website is also used to publish all primary data in accordance with the proposed MIAME standards. Selection of genes for data analysis was based on a greater than 95% probability of being differentially expressed (*P*≤0.05) and a fold change of 2 or greater. The resultant false positive discovery rate was estimated to be less than 0.1% (Walters, unpublished data). We have previously assessed the degree of cross hybridization in chimeric samples and eliminated the small percentage of genes that did cross react from subsequent analysis [43].

### Immunohistochemistry, immunofluorescence and confocal microscopy

*In situ* hybridization using FITC labeled Alu DNA probes (InnoGenex, San Ramon, CA, USA) was performed according to the manufacturer's specifications, and developed using the supersensitive polymer HRP-ISH system (BioGenex). TUNEL was performed using the Apoptag Plus Peroxidase In Situ Apoptosis Detection kit (Chemicon International, Temecula, CA, USA) according to the manufacturer's specifications. The number of Tunel positive nuclei is an average of 15 fields at 200× magnification.

Haematoxylin and eosin, reticulin, Mason's trichrome, and periodic acid/Shiffs staining were performed according to standard procedures [74]. The sections were graded for inflammatory activity and staged for fibrosis according to the modified Batts and Ludwig scoring system [75]. The degree of fatty change was scored as 0 (<5%), 1 (6–33%), 2 (34–66%) or 3 (>66%). Hepatocyte ballooning and macrophages were scored on a scale of 0–4 where 0 is none and 4 is many. The lobular apoptotic body count is an average of 5 fields counted at 100× magnification.

Immunohistochemical and immunofluorescent analysis was performed on 4 µm formaldehyde fixed paraffin embedded sections that were deparaffinized by incubation in xylene for 5 min, followed by sequential rehydration by incubating twice for 3 min in each of 100%, 95%, and 70% ethanol, followed by a 5 min incubation in distilled water. Antigen retrieval was then performed by boiling in pH 6.0 10 mM citrate buffer for 15 min followed by cooling for an additional 15 min.

For immunohistochemical staining with rabbit anti-FAS antibodies (1∶50, Santa Cruz), or rabbit anti-BAX (1∶50, Cell Signaling Technologies), or purified rabbit IgG isotype control, slides were blocked in normal goat serum, washed, incubated with the primary antibodies, washed, incubated with 3% peroxide, and incubated with secondary goat anti rabbit poly-HRP antibodies (Dako Cytomation). The peroxidase was developed using the DAB Plus liquid substrate chromogen system (Dako Cytomation). For staining with goat anti-GRP78/BiP antibodies (1∶50 Santa Cruz) or purified goat IgG isotype control, slides were blocked with normal donkey serum, incubated with primary antibody, endogenous biotin was blocked using the avidin/biotin blocking kit (Vector laboratories), and the signal was amplified using the ABC method (Vector laboratories). The peroxidase was developed as before. Caspase staining was performed as previously described [6]. FAS staining was scored semi-quantitatively where 0 is no staining, 1 (1–25%), 2 (26–50%), 3 (51–75%) and 4 (76–100%). Caspase staining was scored semi-quantitatively as follows: 0 = none, 1 = focal weak, 2 = diffuse weak; 3 = diffuse weak and focal strong; 4 = diffuse strong. GRP78/BiP staining was scored semi-quantitatively where 0 is no staining, 1 (1–15%), 2 (16–30%), and 3 (31–50%). The intensity of the stain was also scored on a scale of 0–3, where 0 is no staining and 3 is intense staining. Since activated activated BAX has a distinct punctate staining that can be easily distinguished form inactive BAX, active and inactive BAX was scored on separate semi-quantitative scales. Inactive Bax was scored in the same manner as BiP, and the scale for activated Bax was 0 is no staining, 1(1–5%), 2 (5–10%), and 3 (10–15%).

For immunofluorescent confocal microscopy, the slides were deparaffinized, the antigens retrieved as before, and blocked as before. Additional blocking using mouse IgG (0.1 mg/ml) for 1 hour, followed by incubation with goat anti mouse-IgG (1 mg/ml) overnight at 4°C was done prior to incubation with mouse anti HCV NS3/4 diluted 1∶50 (TORDJI-22, Abcam), or its isotype control, and one of rabbit anti-FAS, BAX, GADD (Santa Cruz), NF-κB p65 (C-20 Santa Cruz), BCL-xL (Cell Signaling Technologies), or rabbit IgG all diluted 1∶50, or rabbit anti human Albumin diluted 1∶1000 (Dako Cytomation). Slides were blocked with 3% peroxide prior to incubation with goat anti mouse poly HRP and goat anti rabbit Alexa 488 (diluted 1∶100, Molecular Probes, Eugene, OR, USA). The peroxidase was developed using the TSA Plus fluorescence system with tyramide-tetramethyl red (Perkin Elmer). Mounting media (Vectastain-Vector laboratories) contained 1 µg/ml 4,6-diamidino-2-phenylindole (DAPI). For BiP/GRP78, the staining procedure was essentially the same, except slides were blocked with normal donkey serum and avidin/biotin block (Vector laboratories), the primary antibodies were goat anti-GRP78/Bip with the mouse anti-HCV, and the secondary antibodies were donkey anti-goat alexa 488 (Molecular probes), and donkey anti-mouse biotin, followed by avidin-HRP (Vector laboratories). The peroxidase was developed as before.

For co-localization of HCV antigens and TUNEL reactivity, the In Situ Cell Death Detection kit (fluorescein) (Roche) was used, according to the manufacturer's specifications. The incubation with terminal deoxynucleotidyl transferase was carried out prior to incubation with the primary TORDJI-22 antibody. All subsequent steps were carried out as before. Nuclei were stained using DAPI. Confocal microscopy was carried out using a Zeiss scanning LSM510 microscope with the 351 nm, 488, and 543 nm excitation lines, and digital images were collected with a 1 µm optical slice.

### Accession numbers

The accession numbers for the genes/proteins discussed in this manuscript are the following: HCV-H77c AF011751, TNF-α X20910, FAS M67454, FASL U11821, BiP/GRP78 NM_005347, p53 AF307851, CHOP/GADD153 BC003637, BAX NM138763, BCL-Xl Z23115, BCL-2 M14745, NF-κB p65 Z22751, Caspase-8 U60520, Caspase-3 BC016926, CD68 S57235, RIG-I AF038963, IPS-1-Q7Z434, TLR-3 U88879, TRIF AB086380, IL28RA AY129153, CMKOR1 BC008459, IFITM1 J04164, HLA-DRB5 NM_002125, IFIT1 M24594, IFIT2 M14660, B2M AB021288, HLA-A D3219, HLA-B M15470, HLA-F AY253269, HLA-G NM_002127, GBP1 BC002666, BIRC4BP X99699, CXCL11 U66096, CXCL10 X02530, CXCL9 X72755, PSMB9 NM_002800, OAS3 AF063613, OAS1 X04371, OASL AF063611, STAT1 NM_007315, G1P3 BC15603, G1P2 BC009507, IFI44 D28915, IFI27 X67325, ANGPTL4 AF202636, NR4A1 L13740, BDKRB2 S56772, EPO X02157, PPARGC1A AF106698, PCK1 NM_002591, ARG2 D86724, APOA5 AF202889, AVP M25647, CPT1A L39211, MT1A BC029475, FABP5 M94856, RXRA X52773, SREBF1 BC057388, PLIN AB005293, C11orf11 AB014559, APOF L27050, HPX J03048, CYP1A1 BC023019, SAA2 M26152, THRSP Y0809, SCD AF097514, PBP NM_04139, ACSS2 AF263614, GCK AF041014, FDPS J05262, HMGCR NM_000859, CYP51A1 U51685, SQLE D78130, HSD17B6 AF016509, C5 M57729, FTFD1 X69141, RDH16 NM_003708, TNFSF10 U37518, SC4MOL U93162, HMGCS1 NM_002130, IRE1 AF059198, PERK AF110146, ATF6 AB015856, hAAT X01683.

## Supporting Information

Figure S1 Isotype controls for anti-HCV, FAS, and GRP78/Bip antibodies. Liver sections from H77c infected mice were stained using appropriate antibodies (B, D, F) or their isotype controls (A, C, E) as described in Materials and Methods. Panels A and B are serial sections stained using mouse IgG or mouse anti-HCV respectively, while C and D are 4 sections apart and were stained using rabbit IgG or rabbit anti-FAS IgG respectively, and E and F are 7 sections apart and stained using goat IgG or goat anti-BiP respectively.
(9.52 MB TIF)

Figure S2 Immunohistochemistry of FAS expression and TUNEL reactivity. Liver sections from PBS injected (A and D), replication deficient RNA injected (B and E) and HCV H77c infected (C and F) donor matched chimeric mice were stained using rabbit anti-FAS (A–C), developed using the Vecastain ABC kit and counterstained using haematoxylin. Isotype controls were negative and are shown in Supplemental Figure 1C–D. TUNEL (D–F) was performed using the Apoptag Plus Peroxidase In Situ Apoptosis Detection kit and the nuclei were counterstained with methyl green. Magnification ×400.
(5.37 MB TIF)

Figure S3 Isotype controls for anti-BAX antibodies and comparison of Bax expression in an area that is predominantly mouse. Liver sections from H77c infected mice were stained using anti-BAX antibodies (B–D) or its isotype control (A) as described in Materials and Methods. Panels C and D show fields of the liver that consist of mouse cells (C) and one that is predominantly human hepatocytes (D).
(5.70 MB TIF)

Figure S4 Isotype controls for anti-NF-kB antibodies and comparison of NF-κB expression in an area that is predominantly mouse. Liver sections from H77c infected mice were stained using anti-NF-kB antibodies (B–D) or their isotype controls (A) as described in Materials and Methods. Panels C and D show fields of the liver that consist of mouse cells (C) and one that is predominantly human hepatocytes (D).
(4.87 MB TIF)

## References

[1] Alter MJ, Margolis HS, Krawczynski K, Judson FN, Mares A (1992). The natural history of community-acquired hepatitis C in the United States. The Sentinel Counties Chronic non-A, non-B Hepatitis Study Team.. N Engl J Med.

[2] Roberts MS, Angus DC, Bryce CL, Valenta Z, Weissfeld L (2004). Survival after liver transplantation in the United States: a disease-specific analysis of the UNOS database.. Liver Transpl.

[3] Timm J, Roggendorf M (2007). Sequence diversity of hepatitis C virus: implications for immune control and therapy.. World J Gastroenterol.

[4] Bigger CB, Guerra B, Brasky KM, Hubbard G, Beard MR (2004). Intrahepatic gene expression during chronic hepatitis C virus infection in chimpanzees.. J Virol.

[5] Smith MW, Yue ZN, Korth MJ, Do HA, Boix L (2003). Hepatitis C virus and liver disease: global transcriptional profiling and identification of potential markers.. Hepatology.

[6] Walters KA, Joyce MA, Thompson JC, Smith MW, Yeh MM (2006). Host-specific response to HCV infection in the chimeric SCID-beige/Alb-uPA mouse model: role of the innate antiviral immune response.. PLoS Pathog.

[7] Nelson DR (2001). The immunopathogenesis of hepatitis C virus infection.. Clin Liver Dis.

[8] Kanto T, Hayashi N (2006). Immunopathogenesis of hepatitis C virus infection: multifaceted strategies subverting innate and adaptive immunity.. Intern Med.

[9] Bataller R, Brenner DA (2005). Liver fibrosis.. J Clin Invest.

[10] Canbay A, Higuchi H, Bronk SF, Taniai M, Sebo TJ (2002). Fas enhances fibrogenesis in the bile duct ligated mouse: a link between apoptosis and fibrosis.. Gastroenterology.

[11] Mundt B, Wirth T, Zender L, Waltemathe M, Trautwein C (2005). Tumour necrosis factor related apoptosis inducing ligand (TRAIL) induces hepatic steatosis in viral hepatitis and after alcohol intake.. Gut.

[12] Pianko S, Patella S, Ostapowicz G, Desmond P, Sievert W (2001). Fas-mediated hepatocyte apoptosis is increased by hepatitis C virus infection and alcohol consumption, and may be associated with hepatic fibrosis: mechanisms of liver cell injury in chronic hepatitis C virus infection.. J Viral Hepat.

[13] Riordan SM, Skinner NA, Kurtovic J, Locarnini S, McIver CJ (2006). Toll-like receptor expression in chronic hepatitis C: correlation with pro-inflammatory cytokine levels and liver injury.. Inflamm Res.

[14] Zender L, Hutker S, Mundt B, Waltemathe M, Klein C (2005). NFkappaB-mediated upregulation of bcl-xl restrains TRAIL-mediated apoptosis in murine viral hepatitis.. Hepatology.

[15] Robin MA, Demeilliers C, Sutton A, Paradis V, Maisonneuve C (2005). Alcohol increases tumor necrosis factor alpha and decreases nuclear factor-kappab to activate hepatic apoptosis in genetically obese mice.. Hepatology.

[16] Wang Y, Singh R, Lefkowitch JH, Rigoli RM, Czaja MJ (2006). Tumor necrosis factor-induced toxic liver injury results from JNK2-dependent activation of caspase-8 and the mitochondrial death pathway.. J Biol Chem.

[17] Liedtke C, Groger N, Manns MP, Trautwein C (2006). Interferon-alpha enhances TRAIL-mediated apoptosis by up-regulating caspase-8 transcription in human hepatoma cells.. J Hepatol.

[18] Ogawa K, Yasumura S, Atarashi Y, Minemura M, Miyazaki T (2004). Sodium butyrate enhances Fas-mediated apoptosis of human hepatoma cells.. J Hepatol.

[19] Shigeno M, Nakao K, Ichikawa T, Suzuki K, Kawakami A (2003). Interferon-alpha sensitizes human hepatoma cells to TRAIL-induced apoptosis through DR5 upregulation and NF-kappa B inactivation.. Oncogene.

[20] Kim YS, Schwabe RF, Qian T, Lemasters JJ, Brenner DA (2002). TRAIL-mediated apoptosis requires NF-kappaB inhibition and the mitochondrial permeability transition in human hepatoma cells.. Hepatology.

[21] Imose M, Nagaki M, Naiki T, Osawa Y, Brenner DA (2003). Inhibition of nuclear factor kappaB and phosphatidylinositol 3-kinase/Akt is essential for massive hepatocyte apoptosis induced by tumor necrosis factor alpha in mice.. Liver Int.

[22] Hatano E, Brenner DA (2001). Akt protects mouse hepatocytes from TNF-alpha- and Fas-mediated apoptosis through NK-kappa B activation.. Am J Physiol Gastrointest Liver Physiol.

[23] Hatano E, Bennett BL, Manning AM, Qian T, Lemasters JJ (2001). NF-kappaB stimulates inducible nitric oxide synthase to protect mouse hepatocytes from TNF-alpha- and Fas-mediated apoptosis.. Gastroenterology.

[24] Samanta AK, Huang HJ, Bast RC, Liao WS (2004). Overexpression of MEKK3 confers resistance to apoptosis through activation of NFkappaB.. J Biol Chem.

[25] Hara Y, Hino K, Okuda M, Furutani T, Hidaka I (2006). Hepatitis C virus core protein inhibits deoxycholic acid-mediated apoptosis despite generating mitochondrial reactive oxygen species.. J Gastroenterol.

[26] Lee SH, Kim YK, Kim CS, Seol SK, Kim J (2005). E2 of hepatitis C virus inhibits apoptosis.. J Immunol.

[27] Benali-Furet NL, Chami M, Houel L, De Giorgi F, Vernejoul F (2005). Hepatitis C virus core triggers apoptosis in liver cells by inducing ER stress and ER calcium depletion.. Oncogene.

[28] Chou AH, Tsai HF, Wu YY, Hu CY, Hwang LH (2005). Hepatitis C virus core protein modulates TRAIL-mediated apoptosis by enhancing Bid cleavage and activation of mitochondria apoptosis signaling pathway.. J Immunol.

[29] Chan SW, Egan PA (2005). Hepatitis C virus envelope proteins regulate CHOP via induction of the unfolded protein response.. Faseb J.

[30] Tardif KD, Mori K, Kaufman RJ, Siddiqui A (2004). Hepatitis C virus suppresses the IRE1-XBP1 pathway of the unfolded protein response.. J Biol Chem.

[31] Pavio N, Romano PR, Graczyk TM, Feinstone SM, Taylor DR (2003). Protein synthesis and endoplasmic reticulum stress can be modulated by the hepatitis C virus envelope protein E2 through the eukaryotic initiation factor 2alpha kinase PERK.. J Virol.

[32] Lau DT, Luxon BA, Xiao SY, Beard MR, Lemon SM (2005). Intrahepatic gene expression profiles and alpha-smooth muscle actin patterns in hepatitis C virus induced fibrosis.. Hepatology.

[33] Okuda M, Li K, Beard MR, Showalter LA, Scholle F (2002). Mitochondrial injury, oxidative stress, and antioxidant gene expression are induced by hepatitis C virus core protein.. Gastroenterology.

[34] Tang W, Lazaro CA, Campbell JS, Parks WT, Katze MG (2007). Responses of nontransformed human hepatocytes to conditional expression of full-length hepatitis C virus open reading frame.. Am J Pathol.

[35] Fujita N, Sugimoto R, Ma N, Tanaka H, Iwasa M (2008). Comparison of hepatic oxidative DNA damage in patients with chronic hepatitis B and C.. J Viral Hepat.

[36] Chipuk JE, Kuwana T, Bouchier-Hayes L, Droin NM, Newmeyer DD (2004). Direct activation of Bax by p53 mediates mitochondrial membrane permeabilization and apoptosis.. Science.

[37] Fan J, Ren H, Jia N, Fei E, Zhou T (2008). DJ-1 decreases Bax expression through repressing p53 transcriptional activity.. J Biol Chem.

[38] Gong GZ, Jiang YF, He Y, Lai LY, Zhu YH (2004). HCV NS5A abrogates p53 protein function by interfering with p53-DNA binding.. World J Gastroenterol.

[39] Qadri I, Iwahashi M, Simon F (2002). Hepatitis C virus NS5A protein binds TBP and p53, inhibiting their DNA binding and p53 interactions with TBP and ERCC3.. Biochim Biophys Acta.

[40] Majumder M, Ghosh AK, Steele R, Ray R, Ray RB (2001). Hepatitis C virus NS5A physically associates with p53 and regulates p21/waf1 gene expression in a p53-dependent manner.. J Virol.

[41] Otsuka M, Kato N, Lan K, Yoshida H, Kato J (2000). Hepatitis C virus core protein enhances p53 function through augmentation of DNA binding affinity and transcriptional ability.. J Biol Chem.

[42] Kneteman NM, Weiner AJ, O'Connell J, Collett M, Gao T (2006). Anti-HCV therapies in chimeric scid-Alb/uPA mice parallel outcomes in human clinical application.. Hepatology.

[43] Walters KA, Joyce MA, Thompson JC, Proll S, Wallace J (2006). Application of functional genomics to the chimeric mouse model of HCV infection: optimization of microarray protocols and genomics analysis.. Virol J.

[44] Mercer DF, Schiller DE, Elliott JF, Douglas DN, Hao C (2001). Hepatitis C virus replication in mice with chimeric human livers.. Nat Med.

[45] Yanagi M, Purcell RH, Emerson SU, Bukh J (1997). Transcripts from a single full-length cDNA clone of hepatitis C virus are infectious when directly transfected into the liver of a chimpanzee.. Proc Natl Acad Sci U S A.

[46] Hiraga N, Imamura M, Tsuge M, Noguchi C, Takahashi S (2007). Infection of human hepatocyte chimeric mouse with genetically engineered hepatitis C virus and its susceptibility to interferon.. FEBS Lett.

[47] Wagoner J, Austin M, Green J, Imaizumi T, Casola A (2007). Regulation of CXCL-8 (interleukin-8) induction by double-stranded RNA signaling pathways during hepatitis C virus infection.. J Virol.

[48] Saito T, Owen DM, Jiang F, Marcotrigiano J, Gale M (2008). Innate immunity induced by composition-dependent RIG-I recognition of hepatitis C virus RNA.. Nature.

[49] Liberman E, Fong YL, Selby MJ, Choo QL, Cousens L (1999). Activation of the grp78 and grp94 promoters by hepatitis C virus E2 envelope protein.. J Virol.

[50] Hetz C, Bernasconi P, Fisher J, Lee AH, Bassik MC (2006). Proapoptotic BAX and BAK modulate the unfolded protein response by a direct interaction with IRE1alpha.. Science.

[51] Upton JP, Austgen K, Nishino M, Coakley KM, Hagen A (2008). Caspase-2 cleavage of BID is a critical apoptotic signal downstream of endoplasmic reticulum stress.. Mol Cell Biol.

[52] Kim R, Emi M, Tanabe K, Murakami S (2006). Role of the unfolded protein response in cell death.. Apoptosis.

[53] Schapansky J, Olson K, Van Der Ploeg R, Glazner G (2007). NF-kappaB activated by ER calcium release inhibits Abeta-mediated expression of CHOP protein: enhancement by AD-linked mutant presenilin 1.. Exp Neurol.

[54] Hung JH, Su IJ, Lei HY, Wang HC, Lin WC (2004). Endoplasmic reticulum stress stimulates the expression of cyclooxygenase-2 through activation of NF-kappaB and pp38 mitogen-activated protein kinase.. J Biol Chem.

[55] Loo YM, Owen DM, Li K, Erickson AK, Johnson CL (2006). Viral and therapeutic control of IFN-beta promoter stimulator 1 during hepatitis C virus infection.. Proc Natl Acad Sci U S A.

[56] Parone PA, James DI, Da Cruz S, Mattenberger Y, Donze O (2006). Inhibiting the mitochondrial fission machinery does not prevent Bax/Bak-dependent apoptosis.. Mol Cell Biol.

[57] Qadri I, Iwahashi M, Capasso JM, Hopken MW, Flores S (2004). Induced oxidative stress and activated expression of manganese superoxide dismutase during hepatitis C virus replication: role of JNK, p38 MAPK and AP-1.. Biochem J.

[58] Chowdhury A, Santra A, Bhattacharjee K, Ghatak S, Saha DR (2006). Mitochondrial oxidative stress and permeability transition in isoniazid and rifampicin induced liver injury in mice.. J Hepatol.

[59] Venugopal SK, Chen J, Zhang Y, Clemens D, Follenzi A (2007). Role of MAPK phosphatase-1 in sustained activation of JNK during ethanol-induced apoptosis in hepatocyte-like VL-17A cells.. J Biol Chem.

[60] Canbay A, Feldstein A, Baskin-Bey E, Bronk SF, Gores GJ (2004). The caspase inhibitor IDN-6556 attenuates hepatic injury and fibrosis in the bile duct ligated mouse.. J Pharmacol Exp Ther.

[61] Feldstein AE, Canbay A, Angulo P, Taniai M, Burgart LJ (2003). Hepatocyte apoptosis and fas expression are prominent features of human nonalcoholic steatohepatitis.. Gastroenterology.

[62] Chawla-Sarkar M, Lindner DJ, Liu YF, Williams BR, Sen GC (2003). Apoptosis and interferons: role of interferon-stimulated genes as mediators of apoptosis.. Apoptosis.

[63] Scott I, Norris KL (2008). The mitochondrial antiviral signaling protein, MAVS, is cleaved during apoptosis.. Biochem Biophys Res Commun.

[64] Geisler F, Algul H, Paxian S, Schmid RM (2007). Genetic inactivation of RelA/p65 sensitizes adult mouse hepatocytes to TNF-induced apoptosis in vivo and in vitro.. Gastroenterology.

[65] Sun Q, Sun L, Liu HH, Chen X, Seth RB (2006). The specific and essential role of MAVS in antiviral innate immune responses.. Immunity.

[66] Foy E, Li K, Sumpter R, Loo YM, Johnson CL (2005). Control of antiviral defenses through hepatitis C virus disruption of retinoic acid-inducible gene-I signaling.. Proc Natl Acad Sci U S A.

[67] Seth RB, Sun L, Ea CK, Chen ZJ (2005). Identification and characterization of MAVS, a mitochondrial antiviral signaling protein that activates NF-kappaB and IRF 3.. Cell.

[68] Li K, Foy E, Ferreon JC, Nakamura M, Ferreon AC (2005). Immune evasion by hepatitis C virus NS3/4A protease-mediated cleavage of the Toll-like receptor 3 adaptor protein TRIF.. Proc Natl Acad Sci U S A.

[69] Mann EA, Stanford S, Sherman KE (2006). Prevalence of mutations in hepatitis C virus core protein associated with alteration of NF-kappaB activation.. Virus Res.

[70] Choi SH, Park KJ, Ahn BY, Jung G, Lai MM (2006). Hepatitis C virus nonstructural 5B protein regulates tumor necrosis factor alpha signaling through effects on cellular IkappaB kinase.. Mol Cell Biol.

[71] Liao QJ, Ye LB, Timani KA, She YL, Yang XJ (2005). Hepatitis C virus non-structural 5A protein can enhance full-length core protein-induced nuclear factor-kappaB activation.. World J Gastroenterol.

[72] Joo M, Hahn YS, Kwon M, Sadikot RT, Blackwell TS (2005). Hepatitis C virus core protein suppresses NF-kappaB activation and cyclooxygenase-2 expression by direct interaction with IkappaB kinase beta.. J Virol.

[73] Hsu EC, Hsi B, Hirota-Tsuchihara M, Ruland J, Iorio C (2003). Modified apoptotic molecule (BID) reduces hepatitis C virus infection in mice with chimeric human livers.. Nat Biotechnol.

[74] Drury RAB, Wallington EA (1980). Carleton's Histological Technique.

[75] Batts KP, Ludwig J (1995). Chronic hepatitis. An update on terminology and reporting.. Am J Surg Pathol.

