# Supplementary figures and images for: HCV Induces Oxidative and ER Stress, and Sensitizes Infected Cells to Apoptosis in SCID/Alb-uPA Mice
Source: PLoS Pathog. 2009 Feb 6;5(2):e1000291. doi: 10.1371/journal.ppat.1000291 (PMC2647842; doi:10.1371/journal.ppat.1000291)

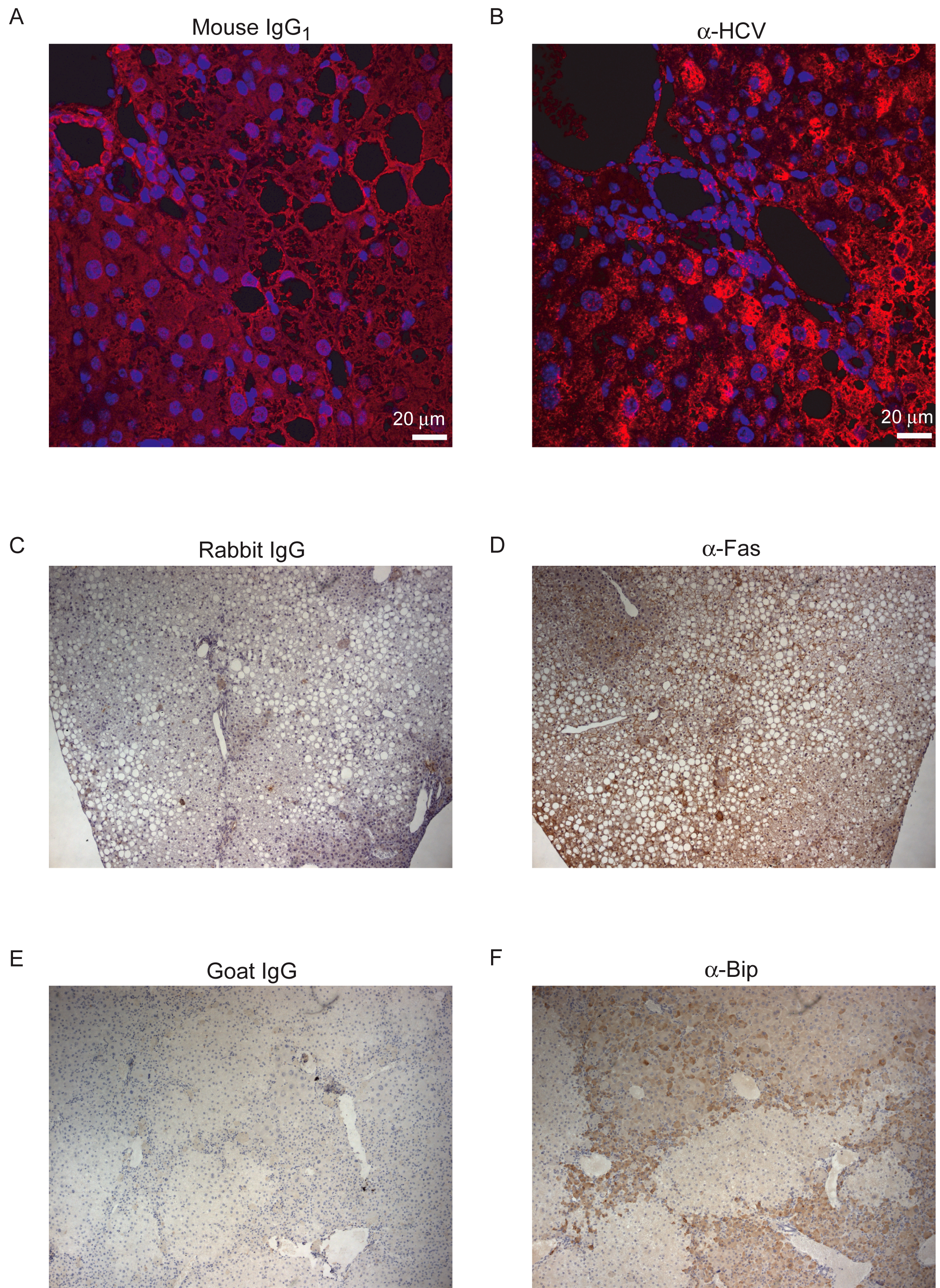

Supplement: Figure S1 — Isotype controls for anti-HCV, FAS, and GRP78/Bip antibodies. Liver sections from H77c infected mice were stained using appropriate antibodies (B, D, F) or their isotype controls (A, C, E) as described in Materials and Methods. Panels A and B are serial sections stained using mouse IgG or mouse anti-HCV respectively, while C and D are 4 sections apart and were stained using rabbit IgG or rabbit anti-FAS IgG respectively, and E and F are 7 sections apart and stained using goat IgG or goat anti-BiP respectively. (9.52 MB TIF) [file ppat.1000291.s001.tif]

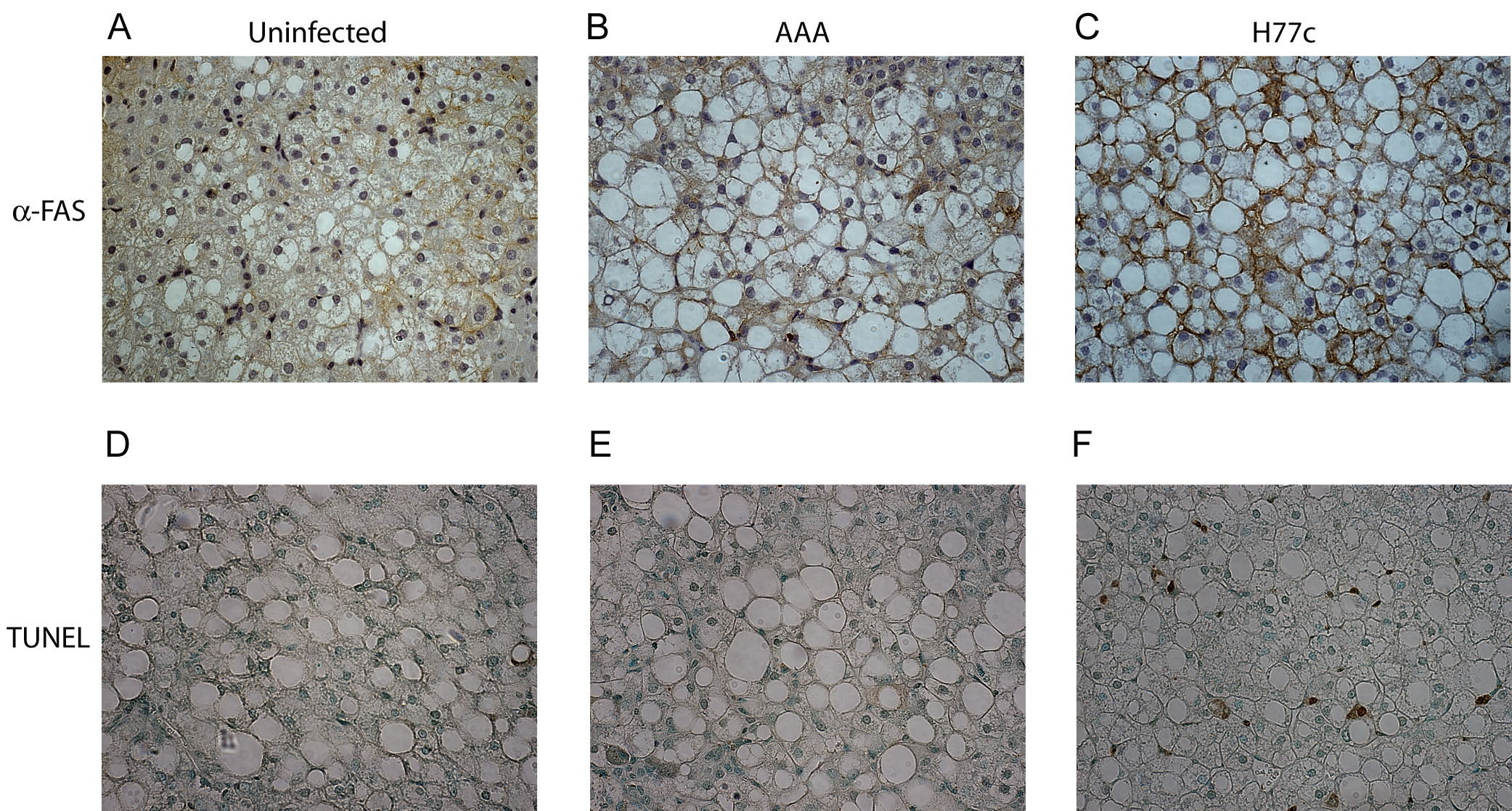

Supplement: Figure S2 — Immunohistochemistry of FAS expression and TUNEL reactivity. Liver sections from PBS injected (A and D), replication deficient RNA injected (B and E) and HCV H77c infected (C and F) donor matched chimeric mice were stained using rabbit anti-FAS (A–C), developed using the Vecastain ABC kit and counterstained using haematoxylin. Isotype controls were negative and are shown in Supplemental Figure 1C–D. TUNEL (D–F) was performed using the Apoptag Plus Peroxidase In Situ Apoptosis Detection kit and the nuclei were counterstained with methyl green. Magnification ×400. (5.37 MB TIF) [file ppat.1000291.s002.tif]

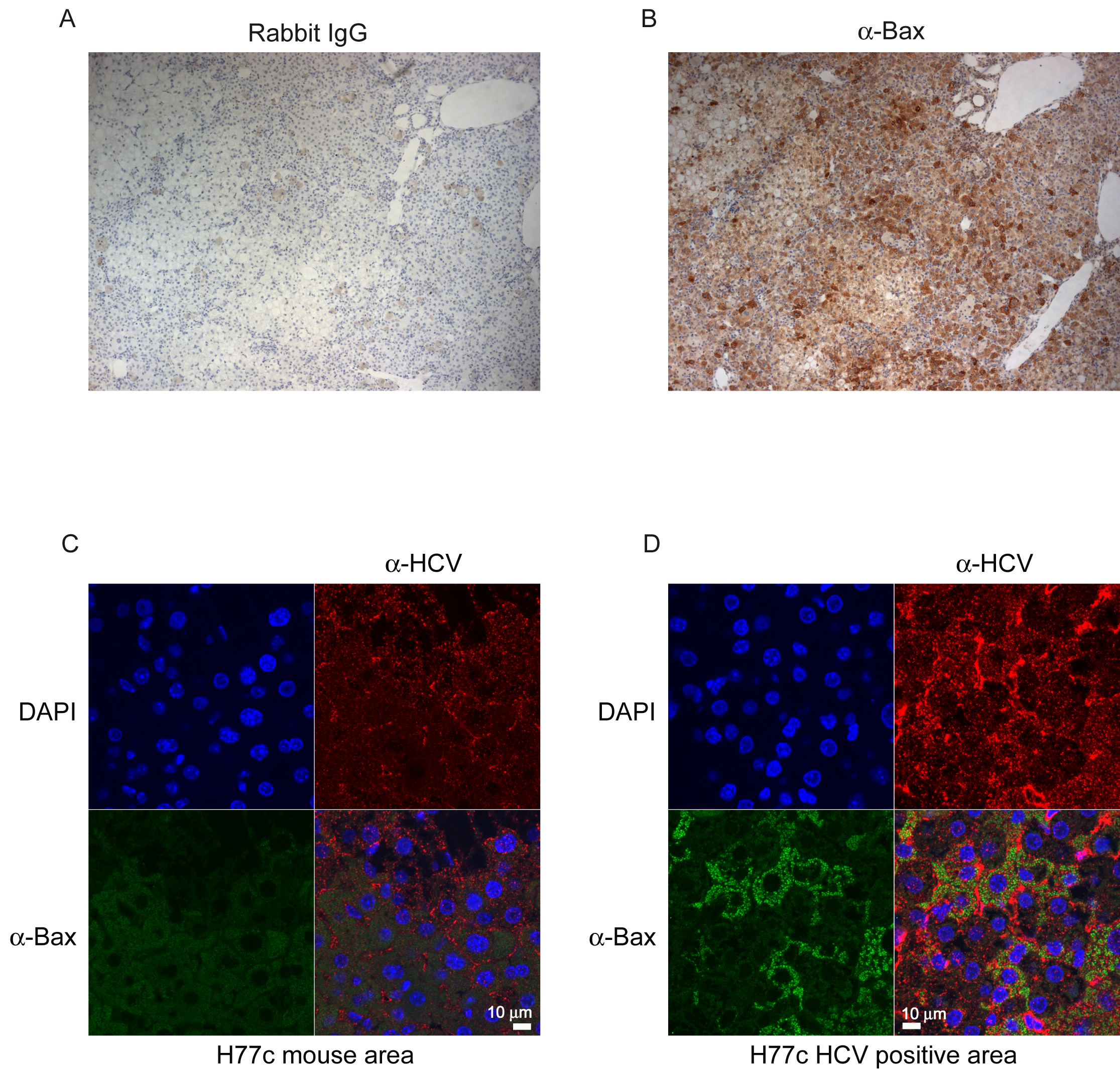

Supplement: Figure S3 — Isotype controls for anti-BAX antibodies and comparison of Bax expression in an area that is predominantly mouse. Liver sections from H77c infected mice were stained using anti-BAX antibodies (B–D) or its isotype control (A) as described in Materials and Methods. Panels C and D show fields of the liver that consist of mouse cells (C) and one that is predominantly human hepatocytes (D). (5.70 MB TIF) [file ppat.1000291.s003.tif]

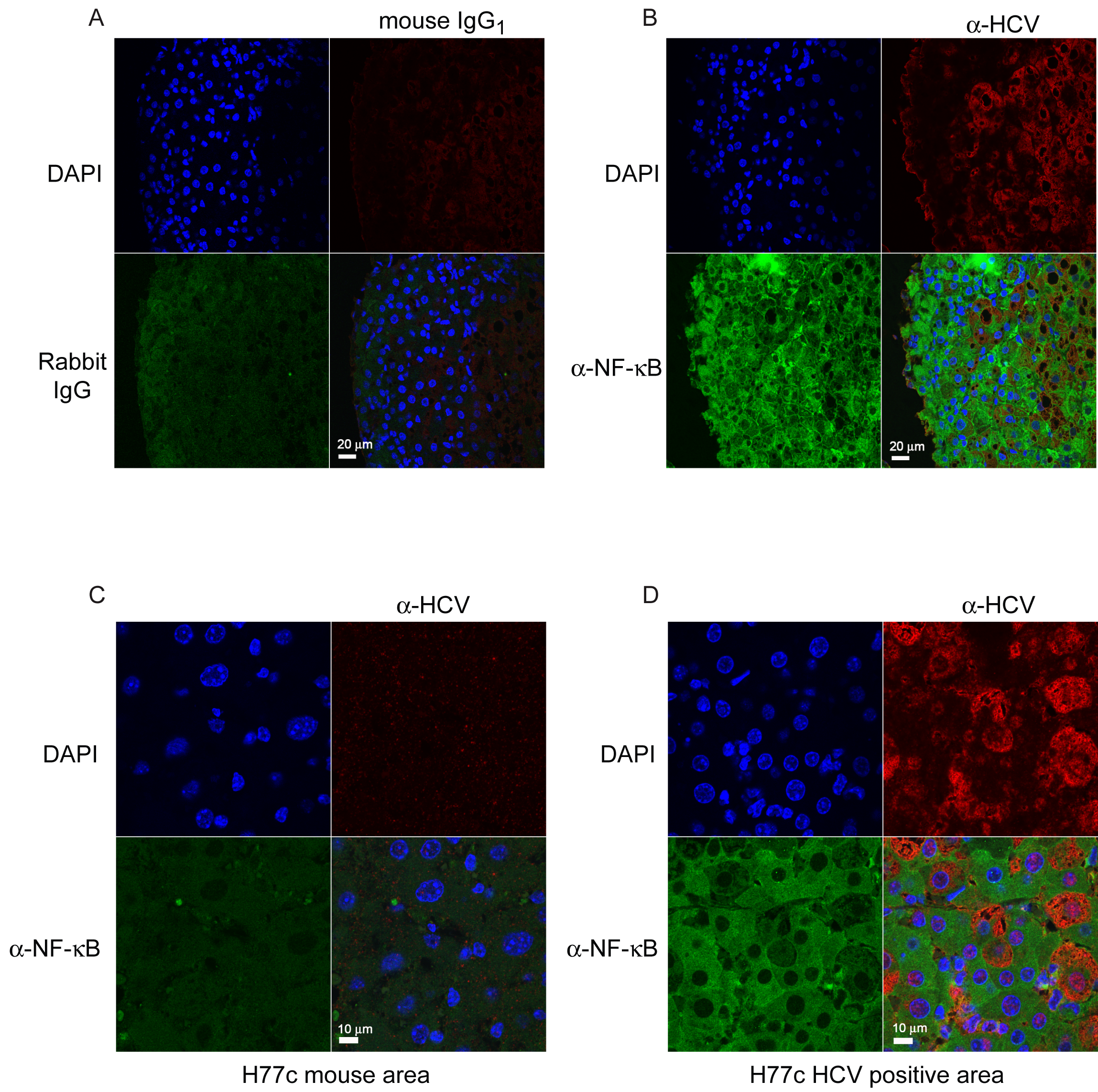

Supplement: Figure S4 — Isotype controls for anti-NF-kB antibodies and comparison of NF-κB expression in an area that is predominantly mouse. Liver sections from H77c infected mice were stained using anti-NF-kB antibodies (B–D) or their isotype controls (A) as described in Materials and Methods. Panels C and D show fields of the liver that consist of mouse cells (C) and one that is predominantly human hepatocytes (D). (4.87 MB TIF) [file ppat.1000291.s004.tif]
